# Equipping AI-decision-support-systems with emotional capabilities? Ethical perspectives

**DOI:** 10.3389/frai.2024.1398395

**Published:** 2024-05-31

**Authors:** Max Tretter

**Affiliations:** Faculty of Humanities, Social Sciences, and Theology, Friedrich-Alexander-Universität Erlangen-Nürnberg, Erlangen, Germany

**Keywords:** emotional intelligence, agency, responsibility, trust, emotion detection

## Abstract

It is important to accompany the research on Emotional Artificial Intelligence with ethical oversight. Previous publications on the ethics of Emotional Artificial Intelligence emphasize the importance of subjecting every (possible) type of Emotional Artificial Intelligence to separate ethical considerations. That’s why, in this contribution I will focus on a particular subset of AI systems: AI-driven Decision-Support Systems (AI-DSS), and ask whether it would be advisable from an ethical perspective to equip these AI systems with emotional capacities. I will show, on one hand, equipping AI-DSS with emotional capabilities offers great opportunities, as they open the possibility to prevent emotionally biased decisions – but that it also amplifies the ethical challenges already posed by emotionally-incapable AI-DSS. Yet, if their introduction is accompanied by a broad social discourse and prepared by suitable measures to address these challenges, I argue, nothing should fundamentally stand in the way of equipping AI-DSS with emotional capabilities.

## 1 Introduction

Emotional Artificial Intelligence (EAI) is a vibrant field of research (McStay, 2018; Misselhorn, 2021; Assunção et al., 2022). One of the main challenges in this field involves crafting AI-systems that are capable of analyzing human gestures, facial expressions, postures, speech, or behavior, and use this biometric data to accurately identify people’s emotional states. This involves interpreting subtle biometric signals, including minor muscle movements or slight variations in vocal pitch, which may signal a range of emotions from stress and happiness to fear and sarcasm. Algorithms with the capability for such nuanced emotion detection are being researched across a range of settings, including *healthcare* (where EAI can be used to improve practitioner-patient interactions (Vagisha and Harendra, 2023) or mental health care (Joshi and Kanoongo, 2022)), *automotive safety* (where EAI is intended to detect signs of drowsiness or distraction and take safety measures (McStay and Urquhart, 2022)), or *education* (where attempts are being made to use EAI to improve pedagogical methods and respond better to the affective states of pupils (McStay, 2020a)). Further, in the realm of social robotics, there’s ongoing research aimed at equipping robots with emotional capabilities, thereby enhancing their ability to engage empathetically and socially with humans (Marcos-Pablos and García-Peñalvo, 2022).

In light of the advancements achieved in the field of EAI over the recent years, along with the vast potential applications of emotionally-capable AI systems and the promising opportunities they offer, a number of studies have emerged offering the ethical perspectives on EAI (McStay, 2018, 2020b; Greene, 2020; Gremsl and Hödl, 2022; Ghotbi, 2023; Gossett, 2023). These investigations have highlighted the potential benefits of emotionally-capable AI systems, while also drawing attention to the associated risks, with key concerns including issues related to privacy, the potential for manipulation, and the threat of exacerbating socio-economic disparities. One central claim found in several ethical discussions on EAI is that the ethical evaluation of this technology hinges on its application context (e.g., healthcare, safety, or advertising) and its intended purpose (e.g., mitigating mental health issues, surveilling public areas, or boosting sales metrics) (Greene, 2020; Ghotbi, 2023).

Against this backdrop, I will focus on one specific type of AI systems: AI-driven Decision-Support-Systems (AI-DSS). These are algorithmic systems typically used in complex decision-making scenarios to analyze these situations with AI, including machine learning and predictive analytics, to deepen understanding, predict potential outcomes of various decision options, and offer data-driven recommendations to facilitate the decision-making process (Phillips-Wren, 2013). I will explore and ask whether it would be advisable from an ethical perspective to equip these AI-systems with emotional capacities. Despite the existence of a significant corpus of research that provides ethical perspectives on AI-DSS in general or their use in specific contexts (Braun et al., 2020; Lara and Deckers, 2020; Stefan and Carutasu, 2020; Cartolovni et al., 2022; Nikola et al., 2022), alongside a comprehensive body of literature addressing the ethics of EAI (McStay, 2018, 2020b; Greene, 2020; Gremsl and Hödl, 2022; Ghotbi, 2023; Gossett, 2023), so far, there has been no research that intersects these two domains. Specifically, there’s a lack of investigation into the ethics of emotionally capable AI-DSS.

My goal is to bridge this gap and to argue that, on one hand, equipping AI-DSS with emotional capabilities offers great opportunities, as they open the possibility to prevent emotionally biased decisions, but that it also amplifies the ethical challenges already posed by emotionally-incapable AI-DSS. Yet, if their introduction is accompanied by a broad social discourse and prepared by suitable measures to address these challenges, I argue, nothing should fundamentally stand in the way of equipping AI-DSS with emotional capabilities.

To substantiate my thesis, I will first focus on the decision-making process, its complexities, and how AI-DSS can assist in making decisions. I will then examine the opportunities and risks associated with equipping these AI-DSS with emotional capabilities, discussing them, and making some suggestions about the advisability of emotionally-capable AI-DSS.

## 2 The difficulty of making decisions and the help of AI

Some decisions are easy to make. Others, however, are difficult. The level of difficulty often hinges on the number of people impacted and the potential severity of the outcomes. Decisions with minimal consequences that affect mainly oneself, such as choosing which pair of shoes to put on in the morning, tend to be simpler than life-altering choices like marriage, which involves other people and bears lasting repercussions. Furthermore, decision-making complexity also escalates with situational complexity and one’s emotional state. A complex situation complicates the clarity of potential outcomes due to information scarcity, challenging the decision-making process (Dewey, 1929; Tretter, 2023). Emotional involvement further exacerbates this challenge, as too much emotion can skew perceptions and introduce biases (Mazzocco et al., 2019; Dorison et al., 2020).

The effects of strong emotional involvement on decision-making can be illustrated using an example from the military sector. Modern military operations are extremely complex and highly dynamic, requiring intricate coordination among various units like infantry, armor, artillery, air support, and logistics to ensure mutual support rather than interference. Furthermore, battlefield conditions can swiftly change, necessitating rapid responses to enemy maneuvers. This complexity and dynamics make strategic decision-making an extremely complicated matter – and can cause continuous emotional stress for the persons in charge. In situations where this stress intensifies, military personnel are more likely to misjudge situations, make a hasty decision, and thereby unduly endanger the lives of those affected (Gamble et al., 2018).

To assist decision-makers in such challenging situations, AI-DSS exist. Provided with sufficient high-quality data, such AI-DSS can quickly comprehend complex situations, analyze them, present possible options, and even simulate the outcomes of various decisions—thus offering recommendations on the most advisable course of action. Such systems are also available, e.g., for the military sector (Scharre, 2020; Szabadföldi, 2021), where AI-DSS are capable of assessing battlefield dynamics in fractions of a second, evaluating the level of threat, and recommending strategies tailored to specific situations. Through such advanced analysis and recommendation processes, AI-DSS significantly bolster the decision-making capacity of military personnel (Liao and Sun, 2020; Horyń et al., 2021).

## 3 The potential of emotionally capable AI-decision-support-systems

As just outlined, AI-DSS can assist in making complex decisions, taking into account a broad array of factors in their analysis, simulations, and advice. At present, however, they are limited by the fact that they cannot take into account the emotional disposition of decision-makers. This oversight is critical because, as demonstrated above, excessive emotional involvement can lead to misperceptions and misjudgments of situations, which in turn may result in hasty or biased decisions.

By equipping AI-DSS with emotional capabilities and enabling them to discern the emotional states of decision-makers, such as military personnel, which exceed the “normal” level of stress associated with such situations and tasks, this shortfall could be remedied. With the ability to assess users’ emotional states, these AI systems could proactively alert individuals if their emotional engagement is likely to impair judgment, making them statistically more prone to errors and biased decisions. In situations where simple alerts might not suffice, the AI could recommend pausing decision-making processes until a more “balanced” emotional state is attained or suggest delegating their responsibilities temporarily. Equipping AI-DSS with emotional capabilities thus offers a forward-looking approach that promises to mitigate the risks of emotionally driven, biased decisions.

It is, no doubt, beneficial to detect and issue warnings about excessive emotional involvement. However, this should not mislead us into believing that emotions are inherently “negative” within the decision-making framework, or that decisions can or should be made on a purely rational basis (Seo and Barrett, 2007). In fact, while over-engagement of emotions can adversely affect decision-making, endeavors to entirely eliminate emotional influence from this process can be just as detrimental. As contemporary research in the field of emotions suggests, there’s a symbiotic relationship between rational thought and emotions, debunking the notion that they are mutually exclusive (Damasio, 1994; Kappelhoff et al., 2019). Given this symbiotic relationship, attempts to exclude emotions from decision-making prove not only unrealistic but also disadvantageous for the decision-making process. This conclusion can be further underscored by everyday observations that, in certain scenarios, emotions can be favorable for decision-making (Mazzocco et al., 2019; Dorison et al., 2020; Gengler, 2020). For instance, worry or fear might prompt more thorough considerations in specific contexts, whereas empathy can lead to decisions that are more compassionate.

The ideal state for decision-making processes involves a “balanced” level of emotional engagement, where decision-makers strike a balance between being excessively emotionally involved and acting like emotionless robots. However, identifying what constitutes a “balanced” degree of emotional engagement in decision-making is complex, as the appropriate level of emotionality significantly varies by context and individual. Ideally, setting “thresholds” for emotional involvement should be personalized and contextual, presenting a substantial challenge. Until tailoring such specific thresholds becomes feasible, employing average benchmarks could serve as a practical interim strategy. This strategy could involve determining the typical degree of emotionality that different individuals demonstrate in specific situations (*situation-specific benchmarks*) or evaluating the general emotional responses of particular individuals across diverse scenarios (*individual-specific benchmarks*). While developing these benchmarks, AI-DSS can be just as useful as in checking, in specific decision-making scenarios, whether decision-makers are too emotionally involved (or not enough).

## 4 The challenges of emotionally capable AI-decision-support-systems

While endowing AI-DSS with emotional capabilities brings significant opportunities, it also raises complex challenges, beginning with the systems’ functionality itself. Current emotionally-capable AI-systems often display biases related to culture, gender, age, and race. This predisposition allows for the precise detection of emotions in white, middle-aged men from Western backgrounds, whereas it fails to recognize with the same accuracy the emotions of individuals from diverse cultures, genders, ages, and racial backgrounds (Shimo, 2020; Kim et al., 2021; Ghotbi, 2023; Gossett, 2023). Yet, even in scenarios where emotionally-capable AI operates flawlessly, recognizing emotions across cultural, gender, age, and racial spectrums without bias, large challenges arise.

Notably, the challenges encountered with emotionally-capable AI-DSS mirror those associated with emotionally-incapable AI-DSS. I will argue that equipping AI-DSS with emotional capabilities exacerbates these existing challenges. In this context, I will particularly focus on the issue of *agency*, and then, building on this foundation, briefly explore the issues of *responsibility*, *accountability*, and *trust*.

One contentious topic in ethical discussions on AI-DSS is the issue of *agency* (Taddeo and Floridi, 2018; Jobin et al., 2019; Braun et al., 2020; Stefan and Carutasu, 2020; Cartolovni et al., 2022; Nikola et al., 2022). While AI-DSS are designed to *support* human decision-making through recommendations, leaving ultimate control with humans, the concern arises that AI’s influence may subtly shift agency away from human decision-makers and toward the AI (Braun et al., 2020). For instance, consider a hypothetical scenario where a physician, despite their instinct or previous experience advocating for a different course of action, may be reticent to question a medical AI system’s treatment suggestion. This reluctance could stem from the perception that the AI system is capable of analyzing a broader array of data, identifying more complex correlations, possessing a more current understanding of medical literature, and executing thorough simulations (Tretter, 2023). This scenario, and similar examples could be found for other contexts, illustrates how difficult it can become for people to contradict the recommendations of AI-DSS and that it may be the easier path to simply agree with AI recommendations. This trend, however, if left unchecked, could gradually erode human agency within the decision-making process.

The hurdles to challenging AI-DSS intensify significantly when individuals, upon deciding against an AI’s recommendation, are subsequently required to justify their decision. In such cases, relying on personal intuition or past experiences may not be considered adequate justification. Confronted with these daunting barriers to overlooking AI suggestions, individuals may increasingly find themselves in a position where they merely validate and approve the proposals of AI-DSS, marking a significant shift in decision-making agency toward AI (Tretter, 2023; Tretter et al., 2023).

Therefore, it is evident that AI-DSS, even without emotional capabilities, can significantly impact user decisions and gradually encroach upon decision-making agency. Incorporating emotional capabilities into AI-DSS may further amplify this issue. Where individuals find themselves having to justify decisions that deviate from AI-generated advice, they now encounter the additional risk that their divergent choices might be attributed to their emotional state. This could further deter people from questioning and deciding against AI recommendations, deepening concerns over the erosion of agency.^1^

Where agency is increasingly challenged by emotionally-capable AI-DSS, this has far-reaching consequences for other issues. If the agency in a decision clearly lies with the human, they can be morally responsible for those decisions and legally liable for their outcomes. However, as humans relinquish more agency, for example, because AI systems significantly influence or even manipulate their decisions or make decisions independently, the less they can legitimately be held responsible and liable. This raises the crucial question of where responsibility and liability should then lie: with the developers of AI-DSS, the institutions that deploy or individuals that use them, the AI system itself, all of them together, or none at all? While such issues of responsibility and liability have been extensively debated in contexts like self-driving cars (Coeckelbergh, 2016; Gless et al., 2016), smart healthcare (Smith, 2021; Sand et al., 2022), and autonomous weapons systems (Santoni de Sio and van den Hoven, 2018; Wood, 2023), no satisfactory resolution has yet emerged. And it is expected that this discussion will become even more complex when AI-DSS are equipped with emotional capacities.

Where responsibility and liability are increasingly called into question by AI-DSS equipped with emotional capabilities, the question arises about the impact this has on existing trust toward these systems. Will trust increase because they can now account for emotional aspects, enabling more thoughtful and sensitive support? Will trust in them decrease due to the heightened risk of unnoticed manipulation by their emotional capabilities? Or will these enhancements have no effect on trust? Further, given that these systems operate within complex sociotechnical frameworks (Schmidl, 2022), the question also arises as to how shifts in trust toward AI-DSS will influence trust toward the domains and institutions deploying them (Samhammer et al., 2023; Tretter et al., 2023).

These concerns about responsibility, liability, and trust are, as hinted above, already relevant in the context on emotionally-incapable AI-DSS. Nevertheless, the extent to which AI systems encroach upon human agency – significantly more so in the case of emotionally-capable AI-DSS than their emotionally-incapable counterparts – amplifies the scrutiny on these follow-up issues. That’s why emotionally-capable AI-DSS intensify these concerns about responsibility, liability, and trust even more.

## 5 Discussion

Considering the opportunities that emerge, alongside the heightened challenges of equipping AI-DSS with emotional capabilities, the question of whether emotionally-capable AI-DSS are ethically advisable cannot be simply answered with a straightforward “yes” or an unequivocal “no.” On one side, dismissing the potential benefits of providing AI-DSS with emotional capabilities by outright rejecting the concept of emotionally-capable AI-DSS would be negligent. Such a choice would ignore the opportunity to mitigate emotionally biased judgments and decisions, potentially risking lives in critical situations (e.g., in the military context).

On the other side, it would be equally negligent to overlook the risks involved and to unconditionally support equipping AI-DSS with emotional capabilities. Opting for this path would fail to address the peril of agency progressively shifting from humans to AI, exacerbating subsequent responsibility gaps, lack of liability, and serious trust issues.

From an ethical perspective, the question of whether AI-DSS should be equipped with emotional capacities might best be answered with a “yes, but….” When a broad societal debate is conducted, in which all perspectives are welcome to deliberate the contexts and manners in which emotionally-capable AI-DSS should be utilized, and if precautionary measures are established from the outset to prevent the loss of human agency, responsibility, and trust, nothing is fundamentally standing in the way of equipping AI-DSS with emotional capabilities. However, this approval remains valid only so long as these stipulations are genuinely fulfilled. Failing to meet these criteria transforms the “yes, but…” into a “no, unless….” As is often the case, the devil lies in the details of execution.

## Data availability statement

The original contributions presented in the study are included in the article/supplementary material, further inquiries can be directed to the corresponding author.

## Author contributions

MT: Writing – review & editing, Writing – original draft.

## Footnotes

^1^Another consideration is that emotionally-capable AI-DSS could tailor their recommendations precisely to the user’s emotional state, allowing them to nudge users toward specific decisions with unparalleled accuracy. The practice of nudging, due to its highly manipulative nature (Sunstein, 2015), remains ethically questionable whether it is carried out by AI-DSS with or without emotional capabilities (Fritzen, 2023). However, the capacity for such nudging is significantly enhanced when employed by emotionally-capable AI-DSS, intensifying the challenge concerning agency.
